# Fabrication of an Optical Fiber Micro-Sphere with a Diameter of Several Tens of Micrometers

**DOI:** 10.3390/ma7074878

**Published:** 2014-06-25

**Authors:** Huijuan Yu, Qiangxian Huang, Jian Zhao

**Affiliations:** 1 School of Instrument Science and Opto-electronics Engineering, Hefei University of Technology, Hefei 230009, China; E-Mail: yuhuijuan@mail.hfut.edu.cn; 2 Product and Technology Department, Hefei Xinsheng Photoelectric Technology Co., Ltd./BOE Technology Group Co., Ltd., Hefei 230009, China; E-Mail: zhaojianhf@boe.com.cn

**Keywords:** optical fiber tapering, Taguchi method, integrated optical fiber micro-sphere, minimum zone method, true sphericity

## Abstract

A new method to fabricate an integrated optical fiber micro-sphere with a diameter within 100 µm, based on the optical fiber tapering technique and the Taguchi method is proposed. Using a 125 µm diameter single-mode (SM) optical fiber, an optical fiber taper with a cone angle is formed with the tapering technique, and the fabrication optimization of a micro-sphere with a diameter of less than 100 µm is achieved using the Taguchi method. The optimum combination of process factors levels is obtained, and the signal-to-noise ratio (SNR) of three quality evaluation parameters and the significance of each process factors influencing them are selected as the two standards. Using the minimum zone method (MZM) to evaluate the quality of the fabricated optical fiber micro-sphere, a three-dimensional (3D) numerical fitting image of its surface profile and the true sphericity are subsequently realized. From the results, an optical fiber micro-sphere with a two-dimensional (2D) diameter less than 80 µm, 2D roundness error less than 0.70 µm, 2D offset distance between the micro-sphere center and the fiber stylus central line less than 0.65 µm, and true sphericity of about 0.5 µm, is fabricated.

## 1. Introduction

Besides being used directly as light transmission components, optical fiber has also been used as optical micro components to satisfy specific functions, by fabricating the fiber ends with certain geometrical shapes. The typical geometrical shapes of fiber ends are tips, lens, spheres, angle ends, side-fire, *etc.* [1], of which the top three types have become hotspots because of their wider range of applications. There are mainly two methods to fabricate fiber ends, namely the laser-heated pulling method and the chemical etching method. Lazarev *et al.* [2,3] used the two methods, respectively, for the formation of near-field scanning optical microscopy tips. Huo *et al.* [4] fabricated several types of optical fiber probe with nano-tips by combining heated micro-pulling and static chemical etching. Concave, convex and spherical are the three types of optical fiber lens tips [5], which would be used for the convergence, divergence and coupling of light, respectively. Shen *et al.* [6,7] performed rapid fabrication of an eyeball-like spherical micro-lens array (ESMA) by extrusion, which can focus light and enhance coupling efficiency. Compared to the combination of optical fiber end and discrete optical components, the fabrication of integrated optical micro components by processing fiber ends directly avoids complicated assembly process, and has the advantages of simple preparation as well as low cost. In addition to the lens, the optical fiber end can also be processed into a sphere for a micro/nano Coordinate Measuring Machine (CMM) probe, which would be used to achieve the true 3D measurement of micro structures with feature size in micrometer magnitude, such as MEMS, precision machinery parts, micro optical elements, *etc.* Especially for contact probes, the geometric dimensions of the probe sphere determine the CMM measurement accuracy to a large extent. Therefore, different from the simple fabrication of optical fiber end for special fiber optical transmission property, the geometric parameters of the optical fiber micro-sphere (in the following section, it will be briefly referred as micro-sphere) must be strictly controlled to a certain level of accuracy.

In order to achieve true 3D measurement of micro devices with a high precision at the micro/nano scale, higher precision of the geometric parameters of the probe sphere is necessary, such as the spherical diameter, sphericity error and offset distance. In addition, simple preparation and low cost are also needed. Several well-known research institutions have done some relevant work, such as PTB [8,9], NIST [10,11] and National Taiwan University (together with Hefei University of Technology) [12,13,14], *etc.* For all of them, how to successfully fabricate a micro-sphere with smaller 2D diameter, 2D roundness error, 2D offset distance between the micro-sphere center and the fiber stylus central line (in the following section, it will be briefly referred to as 2D offset distance) is still the key target. At present, Kuang-chao Fan *et al.* [14] have fabricated a micro-sphere with 2D diameter of about 300 µm, 2D roundness error less than 1 µm and 2D offset distance less than 1 µm. In addition, we have made efforts in the fabrication of the micro-sphere, and a micro-sphere with the spherical diameter of about 100 µm, for which a roundness error less than 2 μm has been obtained [15].

In this paper, utilizing an improved system structure and by the combination of the optical fiber tapering technique and the Taguchi method, a new approach is proposed based on previous work mentioned above to fabricate an integrated optical fiber micro-sphere. Based on the optical fiber tapering technique, several important process factors influencing fabrication quality of the micro-sphere are selected, which include the processing mode of the tapered fiber tip: Here, the cone angle tip is formed in the process of optical fiber tapering. Before the micro-sphere fabrication process by fusion, the tapered fiber tip can be either retained or removed. Other process factors include the distance between optical fiber end and discharge electrode of a fiber fusion splicer, the electric current in the discharge electrode of the fiber fusion splicer, and the discharge duration and the discharge times. All these process factors will be optimized through the Taguchi method. Based on this method, a micro-sphere with a higher level of quality evaluation parameters can be fabricated. It will greatly improve the true 3D measurement ability of micro/nano CMM to micro devices, and this method will provide technical reference for fabrication of optical fiber micro-sphere or micro-ellipsoid with higher quality.

## 2. Results and Discussion

### 2.1. Experiment Principle and Procedure

**Figure 1:**
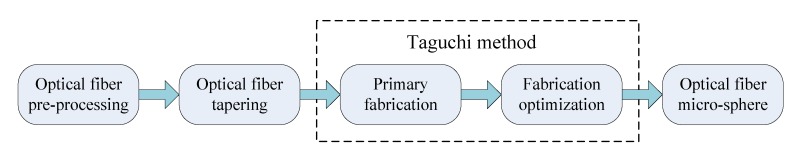
Fabrication process of an integrated optical fiber micro-sphere.

The complete fabrication process of the integrated optical fiber micro-sphere proposed in this paper is shown in Figure 1. After pretreatment, which includes stripping-off of the coating layer and cleaning the surface of the selected optical fiber, a clean bare fiber (consisting of cladding and fiber core only) can be obtained. Through fiber tapering process, the bare fiber is then manufactured to become a fiber taper with a cone angle. Based on a fiber fusion splicer, a fabrication process with two steps is adopted using the Taguchi method, which consists of a primary fabrication process and an optimization fabrication process. Through these processes, a target micro-sphere that meets the requirements can be fabricated successfully.

### 2.2. Optical Tapering

Synthesizing the preliminary fabrication results, it is found that the larger the diameter of the original selected optical fiber, the larger the spherical diameter of the fabricated micro-sphere. That is, an optical fiber with a diameter of 125 µm might generate an ideal micro-sphere with a spherical diameter of above 250 µm. In order to get a smaller ideal micro-sphere, the optical fiber has to be tapered to a very thin diameter before fusion process.

**Figure 2:**
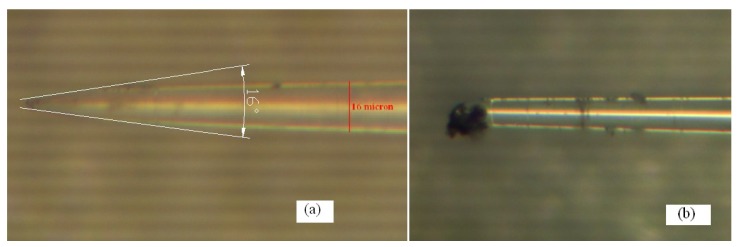
(**a**) Optical fiber taper; (**b**) Fracture tip of a tapered optical fiber.

A finished optical fiber taper is shown in Figure 2. From the Figure 2a, the diameter of the original 125 µm bare fiber is obviously decreased to about ten micrometers, which presents a smooth taper with the cone angle of about 16°. Taking a random cross section near the fiber taper end, it can be seen that the diameter is about 16 µm. Because the tapering principle is based on the bare fiber’s physical properties, and the heating and tapering make the fiber fracture into the fiber taper naturally, thereby the fracture status of the taper tip is an uncontrollable factor. Figure 2b shows a fracture tip of an optical fiber taper. Before the fabrication based on Taguchi method, the processing mode for this fracture tip of the fiber taper, namely keeping removed or retained the taper tip, will be one of the process factors that might influence the subsequent fabrication of the micro-sphere.

### 2.3. Secondary Fabrication Based on the Taguchi Method

#### 2.3.1. Taguchi Method

In order to get the ideal micro-sphere, a fabrication method with two steps is used, of which the first step of primary fabrication is to get a micro-sphere that meets basically the requirements, and the second step of optimization fabrication is to make the quality (geometry parameters) of the fabricated micro-sphere reach the desired accuracy. Due to the particularity of this fabrication method, there are many process factors influencing the fabrication quality. It will cost a lot of time and money if we use a traditional full factional experimental method, so in this paper we use the Taguchi method for the optimization design of the experimental process. The Taguchi method considers three steps in process development: system design, parameter design and tolerance design. In the parameter design step, which is the key of the three steps, the combination of all the parameters for the optimal level is determined, and the key tools are orthogonal array and SNR (signal-to-noise ratio). By choosing appropriate orthogonal array to arrange experiment times, each factor influencing the target characteristics is concluded using fewer experiments. In the orthogonal array, each matrix represents a set of experimental data, and the change of each matrix parameter means the change of its setting value. Actually, these parameters are the influence factors (in the following sections, they will be generally referred to as process factors), while setting values are the levels. Then, the optimum combination conditions and the significance of each process factor are determined according to experimental results with SNR analysis and analysis of variance (ANOVA) [16,17]. In this paper, we firstly use this method to analyze the process factors influencing the quality of fabricated micro-sphere, according to different quality evaluation parameters, then the corresponding optimum combination of process factors is determined, and lastly the optimum combination of process factors to fabricate the optimal micro-sphere is obtained.

**Figure 3:**
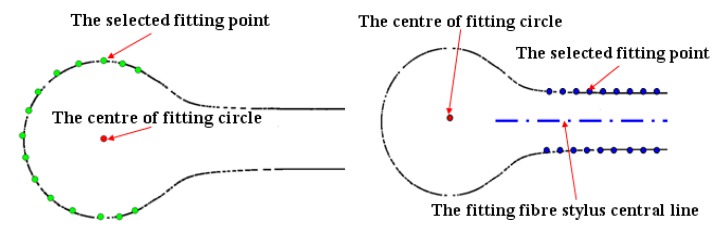
Fitting method of 2D diameter, 2D roundness and 2D offset distance.

The 2D diameter, 2D roundness error and 2D offset distance are selected as the quality evaluation parameters of the fabricated micro-sphere. Compared to the 2D diameter, the last two parameters are more obvious for reflecting the profile and quality of the micro-sphere, so this research focuses on the fabrication process design indexed by the SNR of 2D roundness and 2D offset distance. As for the data acquisition of the three evaluation parameters, due to the small volume of the fabricated micro-sphere and the limitation of the resolution, *etc*., traditional measurement method cannot be applied. Image processing is an effective method [18]. In the experiment, a commercial digital metalloscope (Type ME31) is used for the data acquisition of the fabricated micro-sphere. According to the size of the micro-sphere, we adjust the magnification of the microscope and get the pixel resolution of 0.11 μm/pixel, quantization error of 0.055 μm, which fully meet the measurement requirements. The selected digital microscope camera is MD50 (Guangzhou Ming-Mei Technology Co., Ltd., Guangzhou, China), which employs a real 5 million pixel color CMOS image sensor in progressive scan mode, with resolution of 2592 × 1944 effective pixels. According to calculation, the limit optical resolution of this optical microscope system is about 0.25 µm. Using microscope equipment for image acquisition and processing of the micro-sphere at a different rotation angle, the 2D diameter, 2D roundness error and 2D offset distance are realized. The method diagram is shown in Figure 3. 2D diameter and 2D roundness error are obtained from the fitting circle in 2D cross section using the MZM or the least square method (LSM), and the 2D offset distance is just the distance between the center of fitting circle and the fitting central line of the fiber stylus.

The process factors during fabrication process of the micro-sphere mainly includes the following: the processing mode of fiber taper angle (A), the distance between optical fiber taper tip and the central line of discharge electrodes (B), discharge current in each step (C and D), discharge duration in each step (E and F) and discharge times in each step (G and H). According to experience, except for the first factor, which is considered on two levels; namely, there are two processing modes of the fiber taper angle, which are removed or retained the taper tip. The remaining factors are all considered on three levels. The selected process factors and their levels are shown in Table 1.

In order to select an appropriate orthogonal array, the total degrees of freedom need to be computed. Without considering the factors interaction, the total degrees of freedom is 1 × (2 − 1) + 7 × (3 − 1) = 15. If the original full factorial experimental design was used, it would require 2^1^ × 3^7^ = 4374 trials for all possible combinations of those factors to get the optimum results. We use the Taguchi method, according to the principle that the rows for an orthogonal array should be greater than or at least equal to the total degrees of freedom, an L_18_ (2^1^ × 3^7^) orthogonal array is selected. Obviously, by using the Taguchi orthogonal array L_18_, only 18-group trails are needed to get the optimum factors. Table 2 illustrates the selected orthogonal array L_18_ (2^1^ × 3^7^). Regarding the 2D diameter (not listed in the Table 2), 2D roundness error and 2D offset distance as quality evaluation parameters of the fabricated micro-sphere, 18-group trails experiments are arranged according to experiment conditions listed in the orthogonal array. In order to get reliable experiment results, we repeat actually each group experiment four times, and get experiment results by averaging all the fitting data at 0° cross section.

**Table 1:** Process factors and levels.

Process factors	Processing Mode of Fiber Taper Angle	Distance (Unit)	Discharge Current in Each Step (mA)		Discharge Duration in Each Step (s)		Discharge Times in Each Step (Times)	
Identification	A	B	C	D	E	F	G	H
Level 1	Retained	130	3	1	0.5	0.3	4	2
Level 2	Removed	150	4	1.5	1	0.5	8	3
Level 3	–	170	5	2	1.5	0.7	12	4

**Table 2:** L_18_ (2^1^ × 3^7^) orthogonal array.

L_18_	Process Factors and there Levels								Roundness Error (μm)	Roundness Error SNR	Offset Distance (μm)	Offset Distance SNR
	A	B	C	D	E	F	G	H				
1	1	1	1	1	1	1	1	1	0.94554	1.07309	1.61424	−17.15655
2	1	1	2	2	2	2	2	2	0.91309	1.70455	1.19888	−6.86004
3	1	1	3	3	3	3	3	3	0.91885	1.29227	2.93188	−25.30595
4	1	2	1	1	2	2	3	3	0.74342	5.84103	2.17881	−17.86543
5	1	2	2	2	3	3	1	1	0.93095	1.20897	0.99290	−3.57175
6	1	2	3	3	1	1	2	2	0.97565	0.42203	0.77123	−2.57877
7	1	3	1	2	1	3	2	3	0.84910	3.25091	1.35634	−8.96089
8	1	3	2	3	2	1	3	1	0.84000	3.21805	1.35750	−8.11359
9	1	3	3	1	3	2	1	2	0.83693	3.18562	0.85284	−2.84947
10	2	1	1	3	3	2	2	1	0.84443	3.26853	2.49965	−20.00413
11	2	1	2	1	1	3	3	2	0.68923	7.21080	1.24643	−9.09440
12	2	1	3	2	2	1	1	3	0.82830	3.62569	1.92393	−17.16595
13	2	2	1	2	3	1	3	2	0.71885	6.30348	1.45139	−8.01470
14	2	2	2	3	1	2	1	3	0.80988	4.11467	0.81844	1.41036
15	2	2	3	1	2	3	2	1	0.93278	1.31704	2.19086	−17.01621
16	2	3	1	3	2	3	1	2	1.05155	−1.59014	1.46458	−8.41189
17	2	3	2	1	3	1	2	3	0.81173	3.90608	2.01879	−14.39459
18	2	3	3	2	1	2	3	1	0.90310	1.99284	1.56775	−10.14783

As the evaluation standard which indicates the importance of each process factor, the SNR determines the robustness of the system. In other words, it represents the sensitivity of the system to noise. The larger SNR indicates the smaller sensitivity, and the better robustness [19,20]. In the Taguchi method, the combination of the SNR with highest levels is the optimum combination of all process factors. The experiment’s purpose is to fabricate a micro-sphere with lower spherical diameter, sphericity error and offset distance. Namely, the 2D diameter, 2D roundness error and 2D offset distance should be as small as possible. So in this paper the “smaller-the-better” quality characteristic [21,22] is selected. The “smaller-the-better” means that the value of the parameter is as small as possible, and the extreme value is 0. At this situation, the SNR calculation formula is:

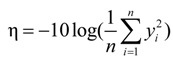
(1)
where η is the SNR calculated by experiment results, *y_i_* is the output response value of the *i*th experiment and *n* is the number of repetitions under the same experimental conditions, in this paper *n* = 4. In the verification experiment, if the error between the SNR of experiment results and the SNR of the desired is less than 10% or 15%, it can be thought that the experiment and desired results are consistent approximately.

The ANONA [17,23] and significance test are carried out to investigate the effect of each different process factor. Following steps should be carried out.

(1) The sum of total squared residuals *S_T_* = *Q_T_* − *p* where *n* is the test number and *x_i_* is the result each of them.


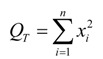
(2)


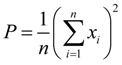
(3)

Taking formulas (2) and (3) into the calculation expression of *S_T_*, the result can be obtained which reflects the total variance of experiment results, and the higher this value means the greater the difference among every experiment results. Two main reasons might result in those differences. One is caused by the levels change of influence factors, and the other is inevitable experiment error. According to the *S_i_* of process factor, we can get the sum of squared residuals for experiment error *S*_error_ as formula (4):

*S*_error_ = *S_T_* + Σ*S*_i_ (4)

(2) Analysis and calculation of the total freedom *f*_total_, the freedom of each process factor *f_i_* and the freedom error *f*_error_.

(3) In order to eliminate the influence of terms number to the sum of squared residuals, it must calculate the average sum of squared residuals from *MS_i_* and their average deviation error *MS*_error_, where:

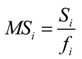
(5)


(4) The significance tests, aiming at estimating each process factor’s significance to expected results. *F* test is used in this paper, and the significant factor α = 0.05. If the calculated F-value of each process factor satisfies 
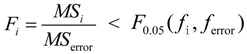
, this factor is not significant, otherwise is significant, and the higher the value, the more significant.

(5) Calculation of the contribution of each process factor to the quality of the fabricated micro-sphere.

#### 2.3.2. Analysis for 2D Roundness Error

**Figure 4:**
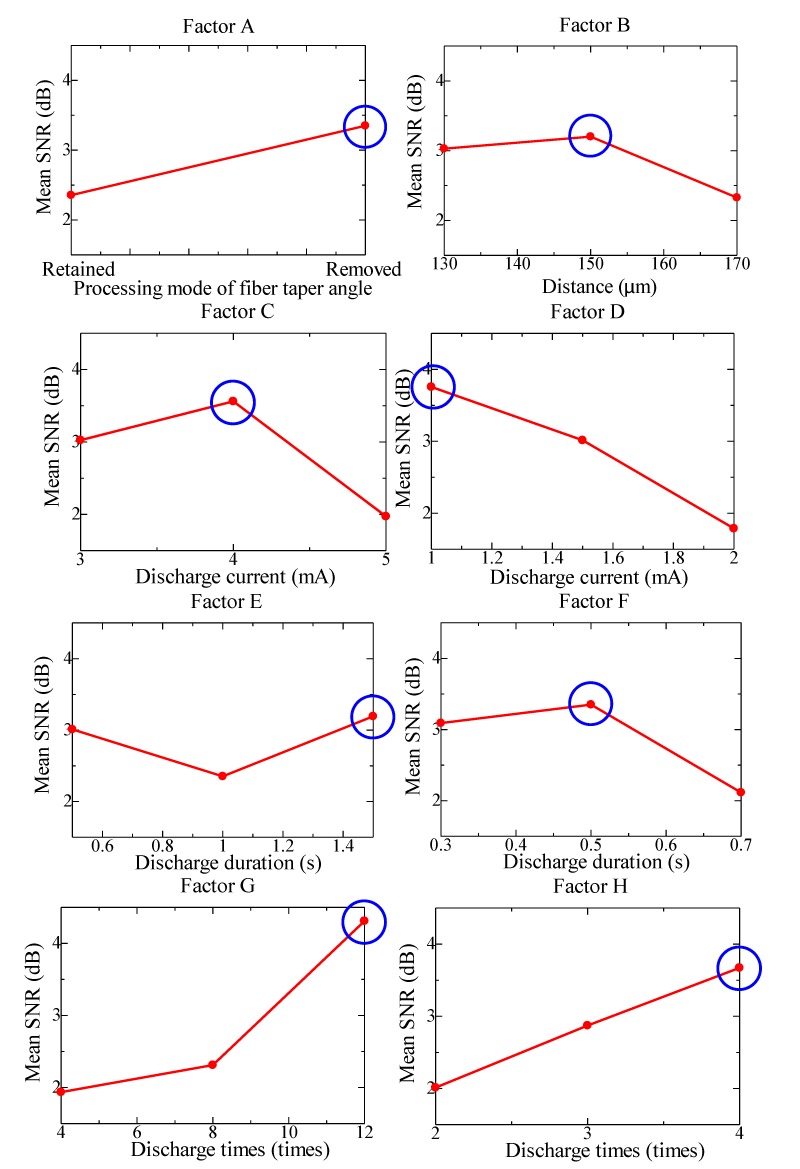
The mean SNR at different levels of process factors for 2D roundness error.

According to the mean SNR of 2D roundness error for each process factor in their different levels, which is shown in Table 2, we get the mean SNR of 2D roundness error responses for each process factor in their different levels, as Figure 4 shows.

According to Taguchi method, the higher SNR indicates the greater impact on the expected result. Thus, from Figure 4, we get the optimum combination of process factors for the 2D roundness error is A2B2C2D1E3F2G3H3. Then, in the condition of the optimum combination, the predicted SNR is as following expression.

η_A2B2C2D1E3F2G3H3_ = η + (η_*A*2_ − η) + (η_*B*2_ − η) + (η_*C*2_ − η) + (η_*D*1_ − η) + (η_*E*3_ − η) + (η_*F*2_ − η) + (η_*G*3_ − η) + (η_*H*3_ − η) = 27.595 − 7 × (2.852528) ≈ 7.63*dB* (6)

where η is the mean SNR for 18-group experiments, η*_xi_* is the SNR of *x* process factor in the *i*th level. It is estimated that the 2D roundness error of the fabricated micro-sphere is 0.6829 μm corresponding to the predicted SNR.

The confirmation experiment is carried out. A micro-sphere is fabricated based on the above optimum combination of the process factors, and the result of 2D roundness error is shown in Table 3.

For the fabricated micro-sphere, the relative deviation between the actual SNR for 2D roundness error and the predicted is

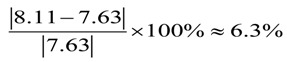
(7)


The result is less than 10%, thus, it is considered that the minimum 2D roundness error could be obtained in the condition of the above predicted optimum combination by Taguchi method.

**Table 3:** Result of 2D roundness error in the condition of optimum combination.

Angle position	0°	45°	90°	135°
2D roundness error (μm)	0.6758	0.69885	0.67389	0.61488
Binary image of optical fibre micro-sphere	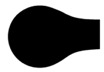	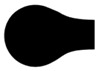	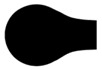	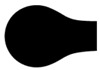
SNR (dB)	8.11			

The result of ANOVA and significance test for 2D roundness error is shown in Table 4. It can be seen that the contribution of each process factor to the quality of fabricated micro-sphere is G>A>H>D>C>F>B>E.

**Table 4:** Result of ANOVA for 2D roundness error.

Factors	SS	DOF	MS	F-value	F_0.05_	Significance **	Contribution (%)
A	6.98	1	6.98	21.8	18.51	YES	16.2
B	5.70	2	2.85	8.9	19.00	NO	6.2
C	8.46	2	4.23	13.2	19.00	NO	9.5
D	12.93	2	6.47	20.2	19.00	YES	15.0
E	3.80	2	1.90	5.9	19.00	NO	3.8
F	8.13	2	4.07	12.7	19.00	NO	9.1
G	21.99	2	11.00	34.3	19.00	YES	26.0
H	13.57	2	6.61	20.7	19.00	YES	15.7
Error	0.64	2	0.32	–	–	–	–
Total	82.2	17	–	–	–	–	–

** At least 95% confidence level.

#### 2.3.3. Analysis for 2D Offset Distance

**Figure 5:**
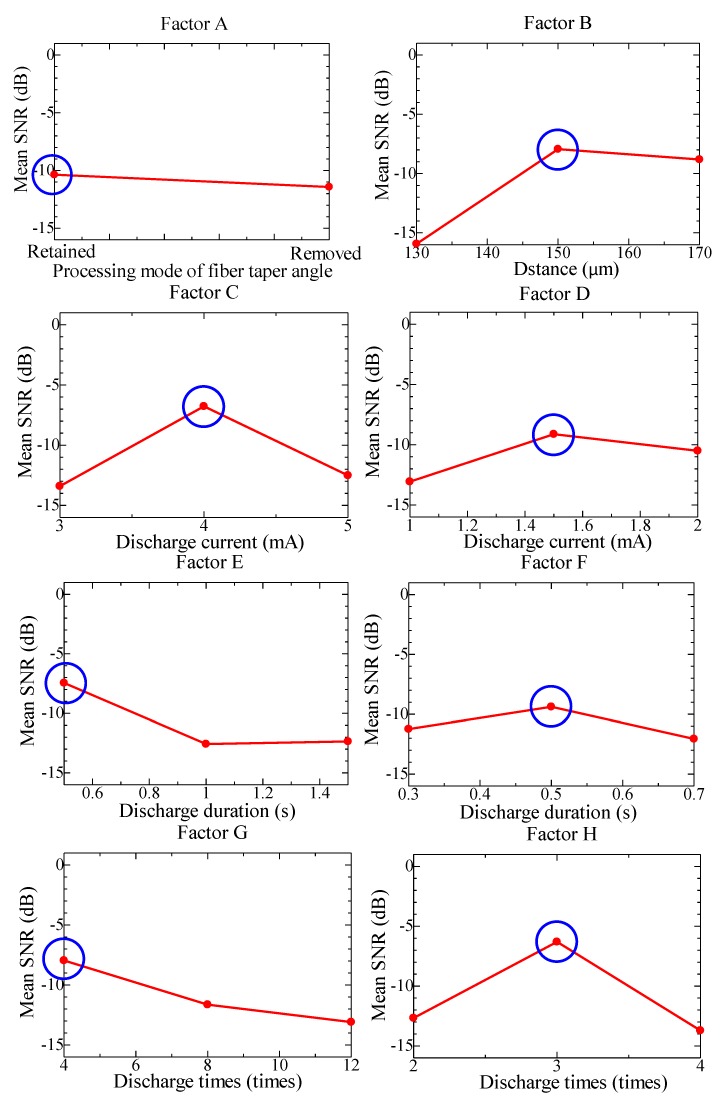
The mean SNR at different levels of process factors for 2D offset distance.

According to the average SNR of 2D offset distance for each process factor in their different levels, which is shown in Table 2, we can get the mean SNR of 2D offset distance responses for each process factor in their different levels, as Figure 5 shows.

According to Taguchi method, from Figure 5 the optimum combination of process factors for the 2D offset distance is A1B2C2D2E1F2G1H2. Then, in the condition of optimum combination, the predicted SNR is as following expression.

η_A1B2C2D3E1F2G1H2_ = η + (η_*A*1_ − η) + (η_*B*2_ − η) + (η_*C*2_ − η) + (η_*D*2_ − η) + (η_*E*1_ − η) + (η_*F*2_ − η) + (η_*G*1_ − η) + (η_*H*2_ − η) = −65.2931 − 7 × (−10.8945) ≈ 10.9684*dB* (8)

where η is the mean SNR for 18-group experiments, η*_xi_* is the SNR of *x* process factor in the *i*th level. It is estimated that the 2D offset distance of the fabricated micro-sphere is 0.5778 μm corresponding to the predicted SNR.

The confirmation experiment based on the above optimum combination of the process factors is carried out, and the result of 2D offset distance is shown in Table 5.

**Table 5:** Result of 2D offset distance in the condition of optimum combination.

Angle position	0°	45°	90°	135°
2D offset distance (μm)	0.4328 (MZM) 0.5058 (LSM)	0.6548 (MZM) 0.6222 (LSM)	0.5085 (MZM) 0.3290 (LSM)	0.5006 (MZM) 0.6598 (LSM)
Binary image of optical fiber microball	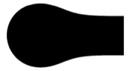	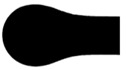	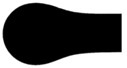	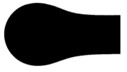
SNR (dB)	12.42			

For the fabricated micro-sphere, the relative deviation between the actual SNR for 2D offset distance and the predicted is

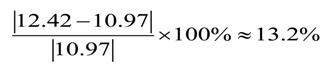
(9)
The result is less than 15%, thus, it is considered that the minimum 2D offset distance could be obtained in the condition of the above predicted optimum combination by Taguchi method.

The result of ANOVA and significance test for 2D offset distance is shown in Table 6. It can be seen that the contribution of each process factor to the quality of fabricated micro-sphere is B>H>C>G>E>D>F>A.

**Table 6:** Result of 2D offset distance in the condition of optimum level combination.

Factors	SS	DOF	MS	F-value	F_0.05_	Significance **	Contribution (%)
A	6.0	1	6.00	1.0	18.51	NO	0
B	258.9	2	122.3	20.38	19.00	YES	28.5
C	160.6	2	80.3	13.38	19.00	NO	17.1
D	49.3	2	24.7	4.12	19.00	NO	4.3
E	62.1	2	31.1	5.18	19.00	NO	5.8
F	28.3	2	14.2	2.37	19.00	NO	1.9
G	90.3	2	45.2	7.53	19.00	NO	9.0
H	199.7	2	99.9	16.65	19.00	NO	21.6
Error	11.9	2	6.0	–	–	–	–
Total	867.1	17	–	–	–	–	–

** At least 95% confidence level.

#### 2.3.4. Analysis for 2D Diameter

Using the same method, we get the optimum combination of process factors for the 2D diameter is A2B3C1D2E1F2G1H2, the relative deviation between the actual SNR for the 2D diameter and the predicted is about 0.3%, which is less than 1%. Thus, it is considered that the minimum 2D diameter could be obtained in the condition of the above predicted optimum combination by Taguchi method, and the contribution of each process factor to the quality of fabricated micro-sphere is C>G>A>B>E>H>F>D.

### 2.4. Quality Evaluation of the Optimum Micro-Sphere

According to the above analysis, we know that, each quality evaluation parameter of the micro-sphere has its optimum combination of process factors respectively, and the contributions of various process factors are different. However, in fact, for the purpose of fabricating a micro-sphere with the best geometric parameters, the selections of these factors are not contradictory. In this research, the main purpose is to fabricate an optical micro-sphere with better sphericity and smaller offset distance, while the absolute size of the spherical diameter is not the focus of attention. Therefore, compared with the spherical diameter, the sphericity error and the offset distance are the two most important parameters for evaluating the quality of the micro-sphere. It means that, under the precondition of a particular 2D diameter, 2D roundness error and 2D offset distance should be seriously considered. On this basis, the optimum combination of process factors that can get all the best evaluation parameters simultaneously could be obtained.

According to the analysis above, based on the Taguchi method, the contribution of each process factor to the spherical diameter (2D diameter) of fabricated micro-sphere is C>G>A>B>E>H>F>D, to sphericity error (2D roundness error) is G>A>H>D>C>F>B>E and to offset distance (2D offset distance) is B>H>C>G>E>D>F>A. Giving priority to the process factors for the sphericity error and offset distance of the fabricated micro-sphere, then we get the final optimum combination of the process factors of A2B2C1D1E1F2G3H2. To be specific, the processing mode of tapered fiber tip is kept removed, the distance between optical fiber end and discharge electrode of a fiber fusion splicer is 150 units, the electric current in the first step is 3 mA, the discharge current in the second step is 1 mA, the discharge duration in first step is 0.5 s, the discharge duration in second step is 0.5 s, the discharge times in first step is 12 and the discharge times in second step is 3.

**Figure 6:**
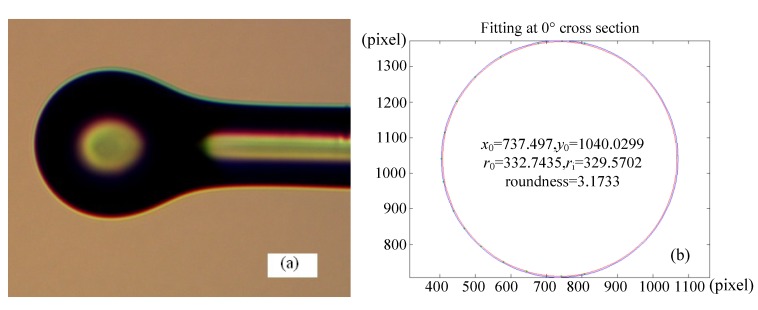
2D cross section of micro-sphere. (**a**) Photography of 2D cross section; (**b**) 2D fitting image.

Verification experiments are done according to the above optimum combination, and high quality micro-spheres are made, of which their 2D diameter are all less than 80 µm, 2D roundness less than 0.7 µm and 2D offset distance less than 0.6 µm. Taking one of the micro-sphere for example, Figure 6a shows the photography of 2D cross section, and Figure 6b is the 2D fitting image based on Matrix Laboratory (MATLAB) software, which is obtained through edge extraction to the 2D cross section by MZM (in the image the numerical unit is pixels). From experiment results, we see that the maximum 2D diameter *D*_max_ = 332.7435 × 2 × 0.11 = 73.20 μm, the minimum 2D diameter *D*_min_ = 329.5702 × 2 × 0.11 = 72.51 μm, the 2D roundness error *R*_error_ = 3.1733 × 0.11 = 0.35 μm and the 2D offset distance *O*_distance_ = abs (1040.0299 − 1045.7714) × 0.11 = 0.63 μm (here, abs means absolute value in MATLAB software).

**Figure 7:**
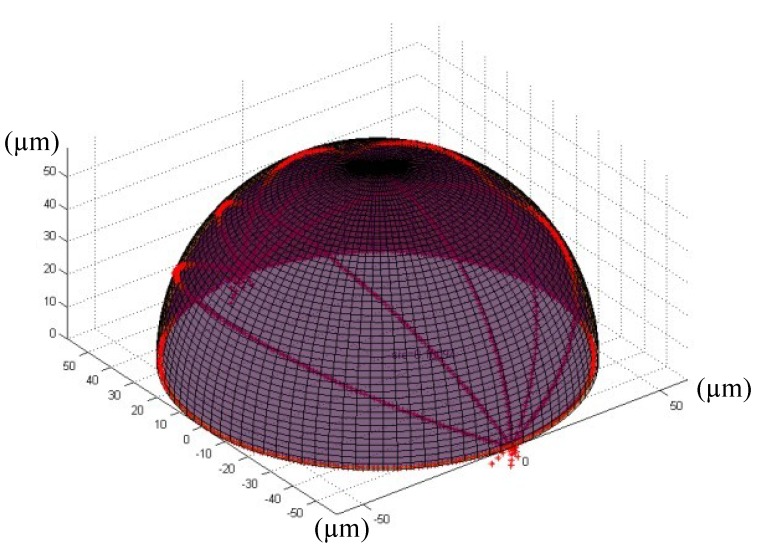
3D fitting surface profile of the optical fiber micro-sphere.

In order to observe the surface profile of the fabricated micro-sphere intuitively, the 3D fitting image of the surface profile is tried out. During the above 2D fitting process, we have already obtained the concentric closed circle of each cross section which contains all the collection points by MZM. If we select different cross sections, groups of concentric closed circle would be obtained, in which the maximum and the minimum are chosen, and be put into a 3D coordinate system that the two concentric closed circle can be extended to two concentric closed balls. Thus, the all collection points of these two sections must also lie between the two concentric closed balls, and then the radius difference between the two balls can be used to evaluate the true sphericity. Evidently, this method conforms to the definition of the true sphericity. The fitting method is that, we converts the 2D edge extraction pixels at different angle position (respectively the section at 0°, 30°, 60°, 90°, 120°, 150° and 180°) into the same 3D coordinate system, and fits the 3D surface profile of the micro-sphere. From the fitting results, the true sphericity can be obtained. Figure 7 is the fitting surface profile of the micro-sphere shown in Figure 6 and it can be found that the true sphericity is 0.51172 μm.

## 3. Experimental Section

### 3.1. Optical Fiber and Fusion Splicer

**Figure 8:**
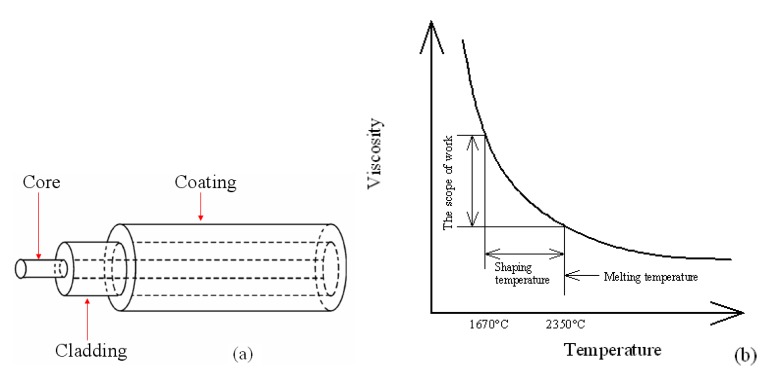
(**a**) Inner structure of the SM optical fiber; (**b**) Relation curve of the optical fiber between temperature and viscosity.

The SMF-28 SM glass optical fiber is selected in this paper. This fiber has a core diameter of 9 µm, a cladding diameter of 125 µm, and a coating layer diameter of 250 µm. The inner structure of the optical fiber is shown in Figure 8a. It consists of two layers of cylindrical medium, in which the inner core is mainly composed of amorphous silica (SiO_2_) while its outer cladding is generally made of glass with high silica content, and the outermost coating layer made of silicone or urethane will be stripped out during the pretreatment process. As previously mentioned, after the pretreatment process of stripping-off the coating layer and cleaning the fiber surface, we get the bare optical fiber, which consists of a cladding layer and the fiber core (the main material is quartz glass). The viscosity of pure vitreous silica is a function of the temperature. As Figure 8b shows, the straining point of the bare fiber (quartz glass) is about 1025 °C and the corresponding viscosity is 10^14.5^ P, the annealing point is about 1120 °C (the corresponding viscosity is 10^13^ P), the softening point is about 1670 °C (the corresponding viscosity is 10^7.6^ P), and the melting temperature range is about 1670–2350 °C (the corresponding viscosity range is 10^4^–10^8^ P), which is just the forming range that the micro-sphere can be fabricated. When the bare fiber is heated up to the molten state, due to the liquid surface tension, the melting quartz fiber liquid contracts to a spherical tip, thus the micro-sphere can be obtained after solidification.

**Figure 9:**
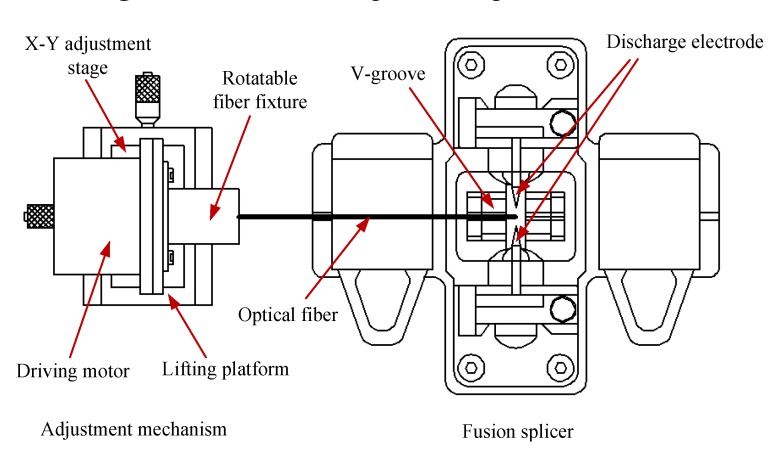
Schematic diagram of experiment device.

**Figure 10:**
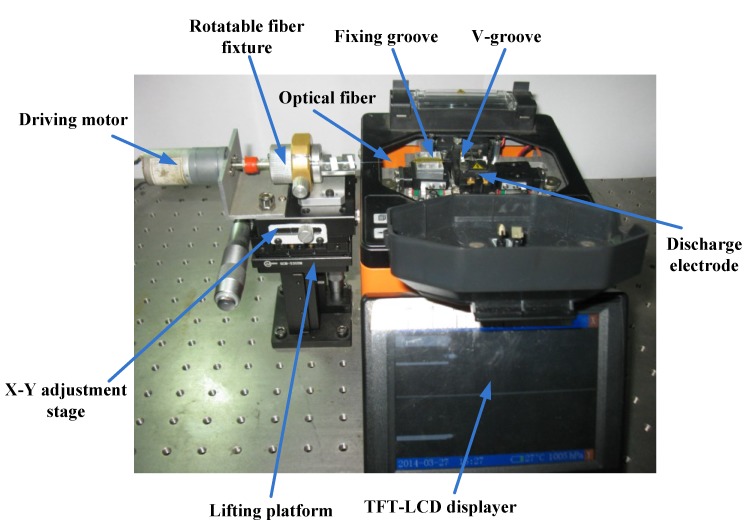
Structure diagram of experiment device.

A commercial optical fiber fusion splicer is employed (Type AV6471) in this experiment. Its basic structure is shown both in Figure 9 and Figure 10 (the splicer part). The locating slot and V-grooves are used to align and hold the bare fiber. The discharge electrodes stimulate an electric arc that heats the bare fiber to the molten state. Tungsten electrodes are used in this fusion splicer with the diameter of 2 mm, electrode angle of 30° and the minimum discharge gap of about 2 mm. The heat generated from electrode discharge can reach about 2000 °C, which is just in the forming temperature range mentioned above, so the bare fiber can be heated to the molten state and the basic requirement of fabrication is satisfied. A TFT-LCD display panel attached to the fusion splicer is shown in Figure 10, which could not only be used to adjust the distance between the bare fiber and the discharge electrodes, but also can achieve online observation of the state of melting optical fiber during the discharge process and the formation process of the micro-sphere.

### 3.2. Experiment Setup and Method

Figure 9 and Figure 10 are the schematic diagram and structure diagram of experiment device for the fabrication of micro-sphere respectively. In order to meet the practical needs of experiment, we improved the original fabrication system without affecting the function of the fusion splicer. That is, in addition to its main structure, adjustment mechanism is added, which contains lifting platform, X-Y adjustment stage, rotatable fiber fixture and driving motor. After the pretreatment and tapering process, we use a rotatable fiber fixture to clamp the processed SM fiber (namely the fiber taper mentioned above), and put the fiber taper exactly into the V-groove of the fusion splicer by adjusting X-Y adjustment stage and lifting platform. Before putting down the cover plate, we use the TFT-LCD display panel to adjust the relative position of the fiber taper to the heated zone resulting from the discharge electrodes. Through setting up reasonable process factors, the heat generated from electric arc between the couple of discharge electrodes is utilized to melt the fiber taper in sintering experiment. During the sintering process, the volume of the micro-sphere tip becomes larger and larger, and due to the self-gravity of the micro-sphere, a certain offset distance between the micro-sphere center and the fiber stylus central line might occur. Therefore, in this paper, we add a driving motor to drive the fiber taper to rotate along with the rotatable fiber fixture with a constant angular velocity (the rotational speed is 110 rpm, and the rotation precision is about ±3°), and the offset distance would be thus reduced or eliminated. In order to do the quality evaluation of the fabricated micro-sphere (the geometry parameters measurement), the 2D image of the micro-sphere is acquired by optical microscopy and fitted through MATLAB software. This has been discussed in the section 2.4.

**Figure 11:**
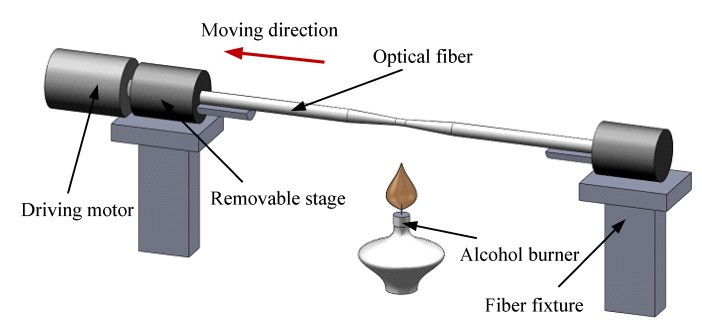
Schematic diagram of optical fiber tapering structure.

Traditional fiber fusion tapering is mainly used to realize the transmission coupling of optical power. It draws closely two optical fibers without coating layer in a certain way firstly, and then stretches on the both sides of the two fibers simultaneously when they are fused during the melting process, finally a double tapered structure can be formed in the heat zone. However, in this paper, our main experimental purpose is to reduce the diameter of the fiber for fabrication of a smaller micro-sphere, so we simplified and improved the traditional system, of which the schematic diagram is shown in Figure 11.

One of the pretreated bare fiber ends is fixed on the optical fiber clamp, namely the fiber fixture on the right side as shown in Figure 11; while the other end is put onto the rotatable stage which is placed on the other fiber fixture. Using an alcohol burner to heat the bare fiber, it can be seen from the relation curve of optical fiber between temperature and viscosity in Figure 8b, that the heated fiber is easy to deform under external forces. Through the driving motor, the bare fiber will be moved left at a precise and constant low linear velocity. During the softening process under evenly heating by the alcohol burner, the bare fiber can be evenly tapered to an ideal fiber taper, of which the cross sectional diameter changes nearly linearly. In the experiment, we use the outer flame of the alcohol burner to heat up the bare fiber, and the positioning accuracy that the bare fiber’s linear movement driven by the driving motor is about 10 µm in magnitude.

## 4. Conclusions

This paper proposed a new method to fabricate an integrated optical fiber micro-sphere based on optical fiber tapering technique and Taguchi method. With the optical fiber tapering technique, a smooth taper with a cone angle of about 16° was obtained. Using the selected orthogonal array L_18_ (2^1^ × 3^7^), and regarding the 2D diameter, 2D roundness error and 2D offset distance as the three quality evaluation parameters, we got the last optimum combination of process factors A2B2C1D1E1F2G3H2 according to the analysis of SNR, ANOVA and significance test, and realized the fabrication with two steps based on the Taguchi method. By using MZM for quality evaluation, an optical fiber micro-sphere with the 2D diameter less than 80 µm, the 2D roundness less than 0.70 µm, the 2D offset distance less than 0.65 µm was fabricated from a 125 µm SM optical fiber. According to the 3D fitting surface profile of the micro-sphere, we can get the sphericity of about 0.5 μm.

In this paper, we propose a new method to fabricate an integrated micro-sphere with small sphericity error, small offset distance and small diameter. However, the integrated micro-spheres with certain diameter are needed in some applications. Therefore, in the next research, some more factors, especially the factors that influence the diameter of the micro-sphere, will be considered, such as the size of the taper cone angle, the stability control of heating method in tapering process, *etc.*
